# Sonification based *de novo* protein design using artificial intelligence, structure prediction, and analysis using molecular modeling

**DOI:** 10.1063/1.5133026

**Published:** 2020-03-17

**Authors:** Chi-Hua Yu, Markus J. Buehler

**Affiliations:** 1 Laboratory for Atomistic and Molecular Mechanics (LAMM), Department of Civil and Environmental Engineering, Massachusetts Institute of Technology, 77 Massachusetts Ave. 1-290, Cambridge, Massachusetts 02139, USA; 2 Department of Engineering Science, National Cheng Kung University, No.1, University Road, Tainan City 701, Taiwan

## Abstract

We report the use of a deep learning model to design *de novo* proteins, based on the interplay of elementary building blocks via hierarchical patterns. The deep neural network model is based on translating protein sequences and structural information into a musical score that features different pitches for each of the amino acids, and variations in note length and note volume reflecting secondary structure information and information about the chain length and distinct protein molecules. We train a deep learning model whose architecture is composed of several long short-term memory units from data consisting of musical representations of proteins classified by certain features, focused here on alpha-helix rich proteins. Using the deep learning model, we then generate *de novo* musical scores and translate the pitch information and chain lengths into sequences of amino acids. We use a Basic Local Alignment Search Tool to compare the predicted amino acid sequences against known proteins, and estimate folded protein structures using the Optimized protein fold RecognitION method (ORION) and MODELLER. We find that the method proposed here can be used to design *de novo* proteins that do not exist yet, and that the designed proteins fold into specified secondary structures. We validate the newly predicted protein by molecular dynamics equilibration in explicit water and subsequent characterization using a normal mode analysis. The method provides a tool to design novel protein materials that could find useful applications as materials in biology, medicine, and engineering.

## INTRODUCTION

The design of hierarchical materials represents one of the frontiers in materials science.^1–3^ In spite of nature's extensive examples of material designs, from silk, to bone, to cells and many others, we are yet to have access to methods that can automatically extract design features from such materials and implement them in new materials that do not yet exist in nature. We propose that the use of machine learning can be a powerful means to extract features and apply neural network models in the design of novel materials. In this paper, we focus specifically on protein materials,^4,5^ which represents an important category of building materials in living systems with important implications for medicine, engineering, and many other fields. Proteins consist of 20 naturally occurring amino acid building blocks that are assembled into hierarchical structures across many length-scales.^5–7^ Examples for protein materials with a structural (e.g., mechanical) function include hair, silk, and tendon. There are many other protein materials with unique optical, biological and tunable, and active properties, for instance materials found in the cell like actin filaments or motor proteins. One way to classify protein materials is by their abundance of the secondary structure, such as alpha-helix, beta-sheet, or random coil. Table I summarizes four different types of protein materials as examples to explain the importance of proteins as the basis for materials design in nature.

**TABLE I.** Summary of different protein materials and their primary structural motif. The focus of this paper is on alpha-helical proteins, which are found widely as the basis of structural materials in nature.

Protein material	Primary structural motif
Hair	Alpha-helices (e.g., keratin protein) forming coiled-coil motifs, cross-linked by disulfide bonds
Intermediate filaments (in cells)	Alpha-helices, assembled as coiled-coils
Spider silk	Beta-sheet mixed with random coil, creating a nanocomposite
Tendon (collagenous tissue)	Triple helix (three amino acid chains forming a rope-like helical structure)

**FIG. 1.**
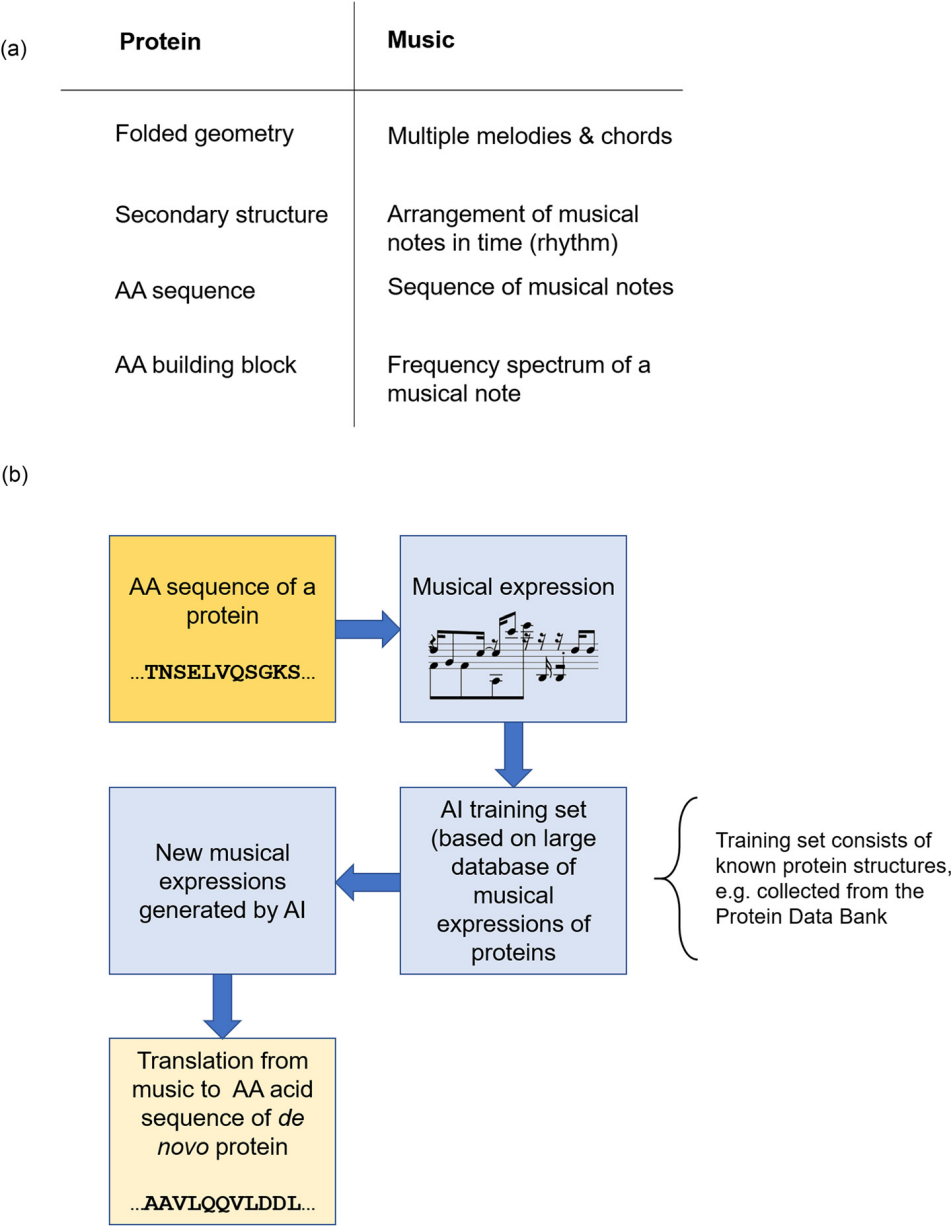
Correspondence of the hierarchical structure of proteins and music (panel a) and overall flow chart of the work reported here (panel b). The approach to reversibly translate between the language of proteins and music allows us to close the loop between different manifestation of hierarchical systems in material and sound, and the reversible translation in between the two representations. New musical expressions generated by human composition offer another pathway to design new materials, albeit it is not yet explored in this paper.

The use of artificial intelligence (AI) in understanding and classifying proteins and predicting new sequences has been explored in recent literature and presents an opportunity for further research investigations.^8–12^ Other work in materials modeling and design has applied AI to design new composites, which can offer an efficient means to materials by design and manufacturing.^13–15^ Here, we apply AI to learn hierarchical structures of protein sequences through a recently proposed model based on long short-term memory (LSTM), one type of recurrent neural network (RNN).^16^ To capture the hierarchical organization of proteins, we exploit an analogy between protein design and music that was proposed earlier by one of the authors.^1,17–20^ This analogy is composed of two major components, the translation of protein structures into musical space and performs design operations in that formulation, and uses a reversible mapping to seamlessly exchange information between these representations. Music represents a similar hierarchical structure as seen in materials design, and may hence be suitable as a mechanism to conduct analysis and design of materials.^21–27^ For instance, protein and music are both made of a limited number of building blocks, which are arranged in particular patterns across scales. Proteins are made of amino acids, and musical pieces are composed of sounds and notes. Both systems feature hierarchical structures: for proteins, all the amino acids are organized in a three-dimensional spatial domain to realize secondary, tertiary folding structures. Musical pieces are created based on different sounding instruments that play certain notes, forming melodies, chords, and other complex structures such as counterpoints in the time domain.^28^ Figure 1 offers a summary view of the method used in this study.

The plan of the paper is as follows. We begin with a description of the translation of existing protein structures into musical scores, used here to develop the training set for our deep neural network. We then provide a description of the deep neural network model and training set, and then present a variety of predicted amino acid sequences, their structures, and further analysis to characterize the designer proteins.

## RESULTS

We now review the results of a variety of amino acid sequence predictions and the resulting protein structures. We note that in the mapping from the musical score back to the amino acid sequence, we solely map the amino acid sequence, and do not capture any secondary or higher-order structural information. This serves as a control mechanism to confirm that the predicted secondary structure, obtained through an analysis of the musical score (specifically, volume and timing of notes), agrees with the predicted protein structures.

The first examples are generated using a small protein with PDB ID 5xdj as a seed, generating 3000 steps. This protein is a small alpha-helix protein consisting of 21 amino acid residues. For a temperature choice close to 1.0, the predicted amino acid sequences match those given in the training set; hence, no *de novo* sequences were generated. For a temperature choice closer to 2.0 and higher, we obtain novel amino acid sequences that are not part of the training set. For example, for a temperature choice of 1.2 with the 5xdj seed, we predict the following sequence (all sequences given in the 1-letter FASTA amino acid code):

GIFSKLAGKKIKNLLISGLKG#GSMKQLEDKVEELLSKNYHL

ENEVARLKKLVGE!GSMKQLEDKVEELLSKNYHLENEVARLK

KLVGE!GSMKQLEDKVEELLSKNYHLENEVARLKKLVG

Note that the symbol # describes the beginning/end of a new protein molecule, and ! describes the end of a protein chain. The first 21 letters reflect the protein that served as the seed, in this case 5xdj. The first part of the sequence was provided by the seed (printed in gray color; the sequence of the prediction always begins with the seed provided). The generated output yields three separate chains, marked in red, green, and black color to separate them. Each of the generated chains consists of the same amino acid sequence. This pattern of creating assemblies of multiple chains with identical sequences is commonly found in naturally occurring proteins, and produced here directly through the neural network prediction. A basic local alignment search tool (BLAST)^37^ analysis reveals that the predicted protein sequence GSMKQLEDKVEELLSKNYHLENEVARLKKLVGE has a 100% identify and 100% query cover with chain A of PDB ID 5iiv, which was included in the training set. A structure prediction of the single chain of the predicted amino acid sequence is shown in Table III.

We now repeat the generation with a higher temperature of 1.4. This results in the following sequence:

GIFSKLAGKKIKNLLISGLKG#GSMTISNMEADMNRLLKQREEL

TKRREKLSKRREKIVKENGEGDKNVANINEEMESLTANIDYIND

SISDCQANIMQMEEAK#DDSEQLQMELKELALEEERLIQ

As before, the predicted sequence begins with the seed printed in gray color, followed by two chains. We analyze the red prediction:

GSMTISNMEADMNRLLKQREELTKRREKLSKRREKIVKENGE

GDKNVANINEEMESLTANIDYINDSISDCQANIMQMEEAK

We find similar as before this protein reflects a protein that exists in the protein data bank, PDB ID 5d3a, which was also included in the training set. A structure prediction of the single chain of the predicted amino acid sequence is shown in Table III.

Once the temperature is set to 1.8 and higher, the predicted sequences show greater variability and yield amino acid sequences that do not exist in the training set or in the protein data bank, hence leading to *de novo* proteins. The prediction for a temperature of 1.8 yields:

GIFSKLAGKKIKNLLISGLKG#SEVELQRALEIARESGTLLA

VVLALEVVARVAIEAARKGNTDAVREALEVALEIARESGTKV

AVVLALEVVARVAIEAARRGNVLAVLALEVVARVAIEAARRG

NVLAVILALEVALEIARESGTE

A BLAST analysis shows that part of the sequence is similar to PDB ID 6e9x. However, the first part of the sequence, specifically the amino acid sequence SEVELQRALEIARESGT, does not match any known sequence. Table III includes the structure prediction result of this small peptide, revealing that it forms an alpha-helix protein. This indicates that the neural network is capable of predicting *de novo* sequences that also feature the characteristics of the class of proteins, in this case, alpha-helix rich proteins.

We now select a temperature choice of 2.0 and generate long musical scores with 10 000 steps length, and translate those back into amino acid sequences. For a seed using the PDB 5xdj protein as for the earlier examples, we predict this sequence:

GIFSKLAGKKIKNLLISGLKG#GSMEKEDKVEELLSKNYHLE

NEVARVKRLRLEVERDNLAEDIMRLREKLQEEKLRRCAVKGR

HQDRCLELTKRNQELEANKKLALTEIEKELEAETRAEFAELS

ALQEEKQRRKVEQQLADKAIQEEKARKCKVEELEAKKKNIDD

LEDKVEENQHRVDGAEKNGMLADVYQAELRRKVEELERALRA

VEEAARLAKRNLLKAVEENQEMLELKEKLSVELLTLAEFAEE

EIDDVEISQLAKKLLGGW!AAVLQQVLDDLAGLCAANIAGGV

LAQVARESSEIQEQIARRAIVLEKQARLLTQLADVKKQQQLG

ALKMIAAEEEERRRRQQQLMNKAIAAKKGNTDDLQKALRTKR

RVREAKKVLSLEEEEVTRLLRIEEEVREEARRKKQLHEAEAG

SARRRQLKMLALLVAEEAARRVAAERVREEARELLEVIRFRA

VELLEVEELLRVLEEEEIQALRNQQYILSVNNNATLARLDLL

AADLLNALLTLLEEEEEERRLLQQQLKIEEEIQL#

This prediction includes two new amino acid sequences, marked in red and green. Based on the BLAST analysis, we find that the first sequence in red does not match any protein in the training set, nor does it match any known existing protein. This sequence:

GSMEKEDKVEELLSKNYHLENEVARVKRLRLEVERDNLAEDI

MRLREKLQEEKLRRCAVKGRHQDRCLELTKRNQELEANKKLA

LTEIEKELEAETRAEFAELSALQEEKQRRKVEQQLADKAIQE

EKARKCKVEELEAKKKNIDDLEDKVEENQHRVDGAEKNGMLA

DVYQAELRRKVEELERALRAVEEAARLAKRNLLKAVEENQEM

LELKEKLSVELLTLAEFAEEEIDDVEISQLAKKLLGGW

A detailed analysis of the BLAST results shows that a 96% query cover leads to 37.97 sequence alignment with PDB ID 2efR. However, in addition to showing only a 37.97% sequence alignment, we note that PDB ID 2efr was not part of the training set. We find that the protein PDB ID 2efr is an alpha helical leucine zipper. The highest sequence alignment is with 5iew but it covers only 14% of the sequence queried, implying that the entire sequence shows a variety of distinct patterns. The structure prediction of this *de novo* sequence is shown in Table III, confirming that this protein is indeed an alpha-helical protein. These results show that the method is capable of generating new proteins from sequences the neural network has learned. Also notable is that the method predicts the correct space signals, i.e., new chains and protein structures separated by # and !. Similarly, we analyze the second sequence predicted:

AAVLQQVLDDLAGLCAANIAGGVLAQVARESSEIQEQIARRA

IVLEKQARLLTQLADVKKQQQLGALKMIAAEEEERRRRQQQL

MNKAIAAKKGNTDDLQKALRTKRRVREAKKVLSLEEEEVTRL

LRIEEEVREEARRKKQLHEAEAGSARRRQLKMLALLVAEEAA

RRVAAERVREEARELLEVIRFRAVELLEVEELLRVLEEEEIQ

ALRNQQYILSVNNNATLARLDLLAADLLNALLTLLEEEEEER

RLLQQQLKIEEEIQL

**FIG. 2.**
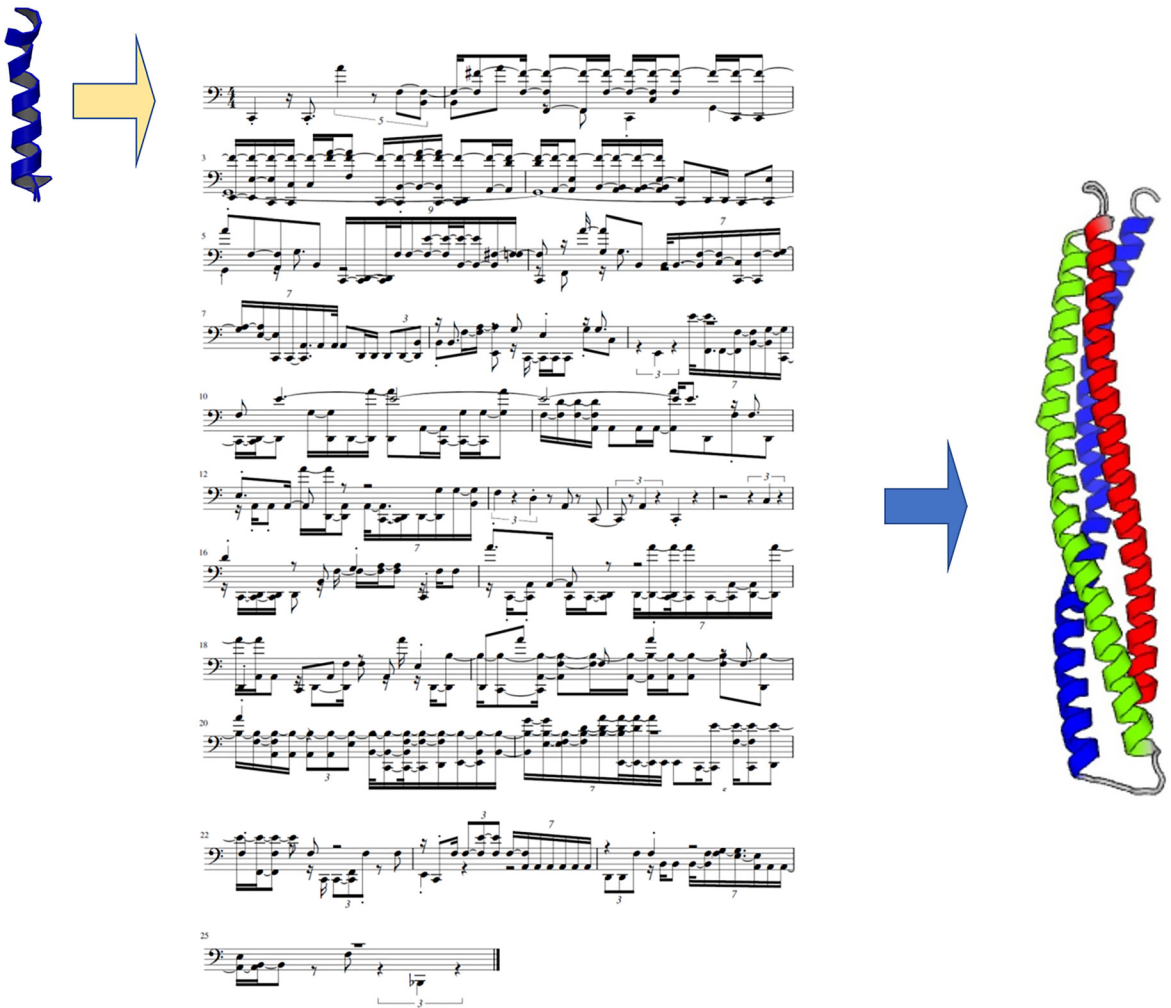
Musical score predicted by the neural network (center), translated into the amino acid sequence, and predicted protein structure (right). This protein does not exist in any database and is completely *de novo*, designed by the neural network. The small protein on the very left is PDB ID 5xdj, used as the seed in the generation process. Note that the sequence of the protein is played sequentially in its entirety.

This is also a *de novo* protein that does not exist in any of the databases. The BLAST analysis shows that for the highest query cover of 97% the sequence alignment is 30.41% with known proteins. The highest sequence alignment is 39.56%, which covers 86% of the query. Figure 2 shows the musical score generated for this case, from which we extracted the green amino acid sequence, as well as the predicted protein structure (the protein structure is the same as already shown in Table III). Analyzing the musical score, we notice that the rhythm and note volumes clearly indicate an alpha-helix secondary structure. The musical score indicates *three* distinct alpha helical segments, created by two breaks of the “helical rhythm,” in agreement with the predicted structure. This indicates that information about a higher-order protein structure can be directly readout of the musical score, as confirmed in the folding prediction.

**FIG. 3.**
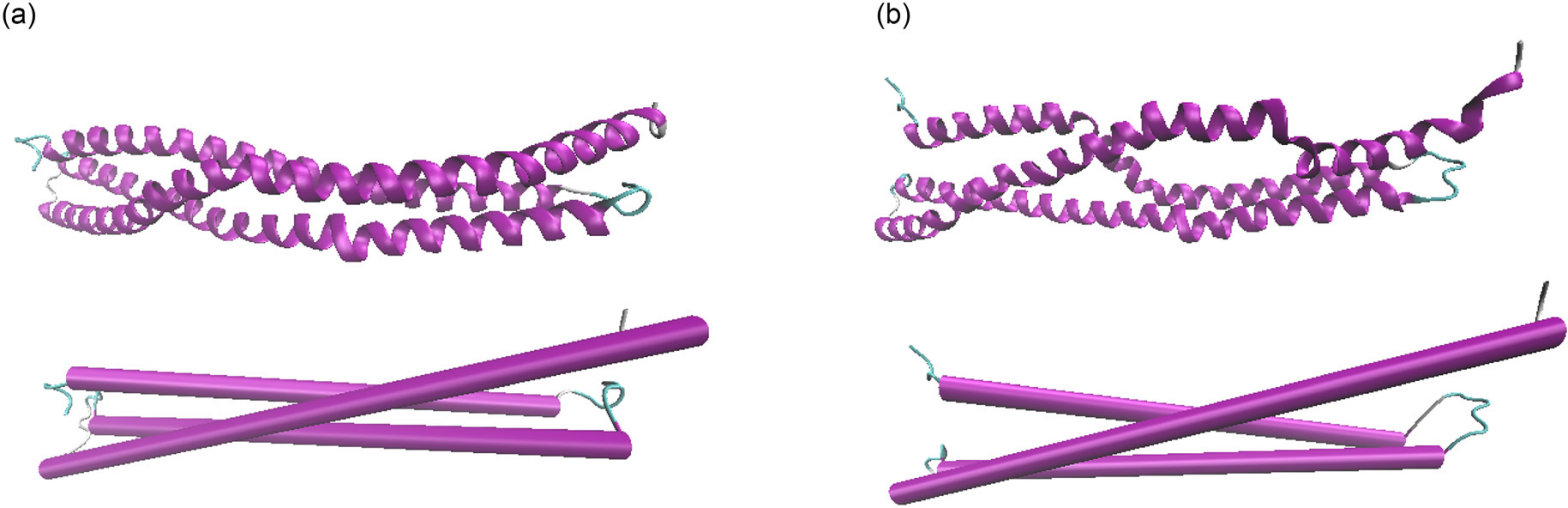
*De novo* protein with one chain equilibrated in explicit water for 3.5 *μ*s. Original structure (panel a) and structure in equilibrium (panel b). The top representation shows the results based on the NewCartoon method, and the lower plots the results using the Cartoon method. The data show that the protein is well equilibrated.

**FIG. 4.**
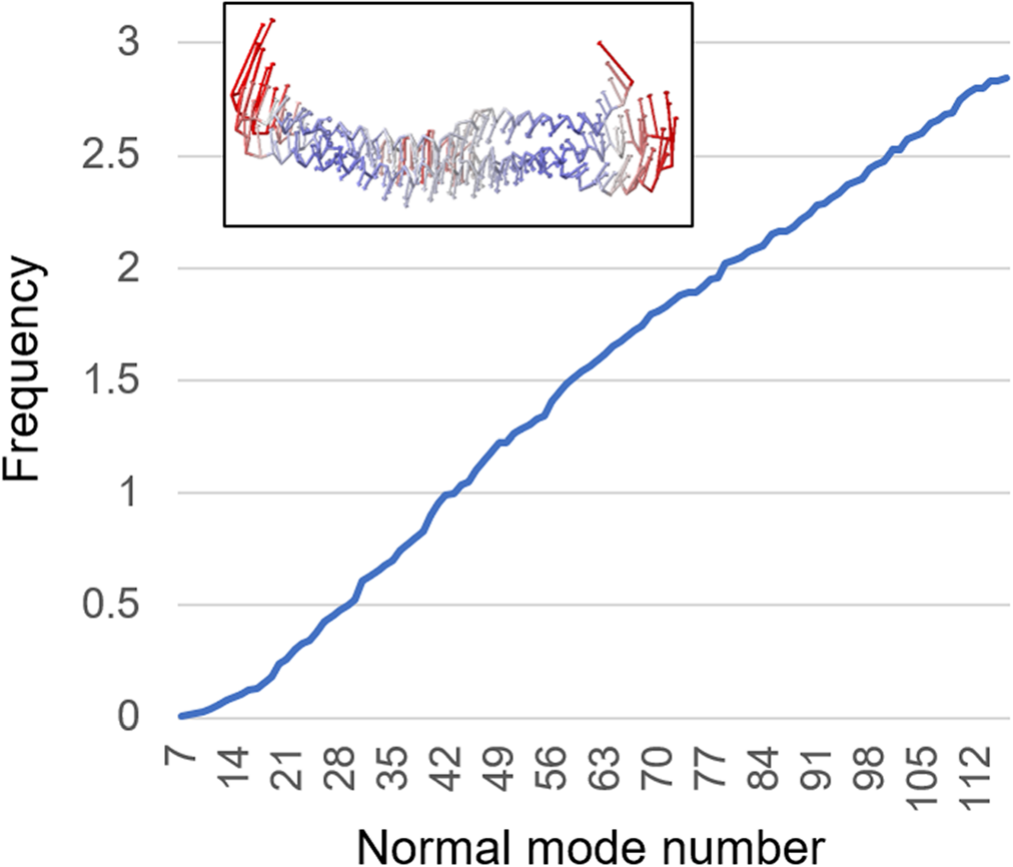
Normal mode analysis of the *de novo* protein, plotting the frequency over the normal mode number. The inset in the plot shows the deformation profile of the protein in mode 1, as an example for nanomechanical deformations induced by the vibrations.

While the ORION and MODELER tools^38,39^ offer a useful way to estimate structures of proteins, to refine the predictions molecular dynamics simulations are required. We exemplify this based on the second sequence reviewed above (the green sequence) and build a molecular dynamics model using CHARMM and explicit solvent, at neutral pH and 0.15 mol/l NaCl concentration. Energy minimization and equilibration confirm that the predicted structure is stable and retains its alpha-helical geometry. Figure 3 depicts the initial and equilibrated protein structure after 3.5 *μ*s in explicit water. Further analysis could be done using Steered Molecular Dynamics (e.g., to determine the Young's modulus of the protein) or other approaches. A simple way to probe the nanomechanical properties of a molecule is using normal mode analysis. Figure 4 shows an example analysis of theprotein, carried out using an anisotropic elastic network model.^46^ To illustrate how this *de novo* protein sounds like in its musical representation, please see PDB1_sonified.mp3 (in the supplementary material).

To illustrate the versatility of the method, we briefly review a few more results. For a seed using PDB 2ndk (human dermcidin, an antimicrobial peptide secreted constitutively by sweat glands), and a temperature of 1.2, we predict this sequence:

SSLLEKGLDGAKKAVGGLGKLGKDAVEDLESVGKGAVHDVKD

VLDSVL#EHEAERRDKNKLTAETEKGIMAYMAFLKEAERRSD

EGQTNTVTLQDLLNVKMALDIEIATYRKLLEG#S

As one can confirm from the gray sequence, the seed amino acid sequence is longer than the previous examples. The predicted sequence marked in red is a combination of a *de novo* sequence at the beginning and a fragment of the vimentin intermediate filament sequence toward the end (the QDLLNVKMALD IEIATYRKLLEG fragment; underlined in the sequence above). The structure predicted based on this sequence is also shown in Table III. It is noted that the structure of the first sequence part alone yields a protein that is not fully alpha-helical, but that consists of an alpha-helix and a random coil segment next to it (structure also shown in Table III). However, when the entire sequence is considered, as predicted by the neural network, a fully alpha helical sequence is formed. This may imply that the method is indeed capable of capturing longer-scale sequence to structure relationships in the generated folds.

## DISCUSSION AND CONCLUSION

In this paper, we reported a new approach to understand protein structures in musical space. This translation may offer new avenues to understand the protein function and how it changes under variations of sequence, secondary structure, and other structural parameters. The deep neural network is capable of training, classifying and generating new protein sequences, ranging from reproducing existing sequences and those included in the training set to completely new sequences that do not exist yet. Unlike most other AI based models that focus mainly on predicting the folding structure, our approach targets generating new proteins with an embedded secondary structure. Our method opens an opportunity to understand patterns in various forms of hierarchical systems and how they can be designed through distinct representations. In general, other sonification approaches to translate proteins into music (different from what we used in this paper) are possible as well, as long as it is unique to enable reversibility.

Proteins are the most abundant materials of all living things. Their motion, structure, and failure in the context of both normal physiological function and disease are a foundational question that transcends academic disciplines. In this paper, we focused on developing a model for the vibrational spectrum of the amino acid building blocks of proteins, an elementary structure from which materials in living systems are built on. This concept is broadly important as at the nano-level observation, all structures continuously move, reflecting the fact that they are tiny objects excited by thermal energy and set in motion to undergo large deformations, which we exploit here to extract new musical compositions as one way to represent nature's concept of hierarchy as a paradigm to create function from universal building blocks. More broadly, the translation from various hierarchical systems into one another poses a paradigm to understand the emergence of properties in materials, sound, and related systems, and offers new design methods for such systems where large-scale and small-scale relationships interplay.

**FIG. 5.**
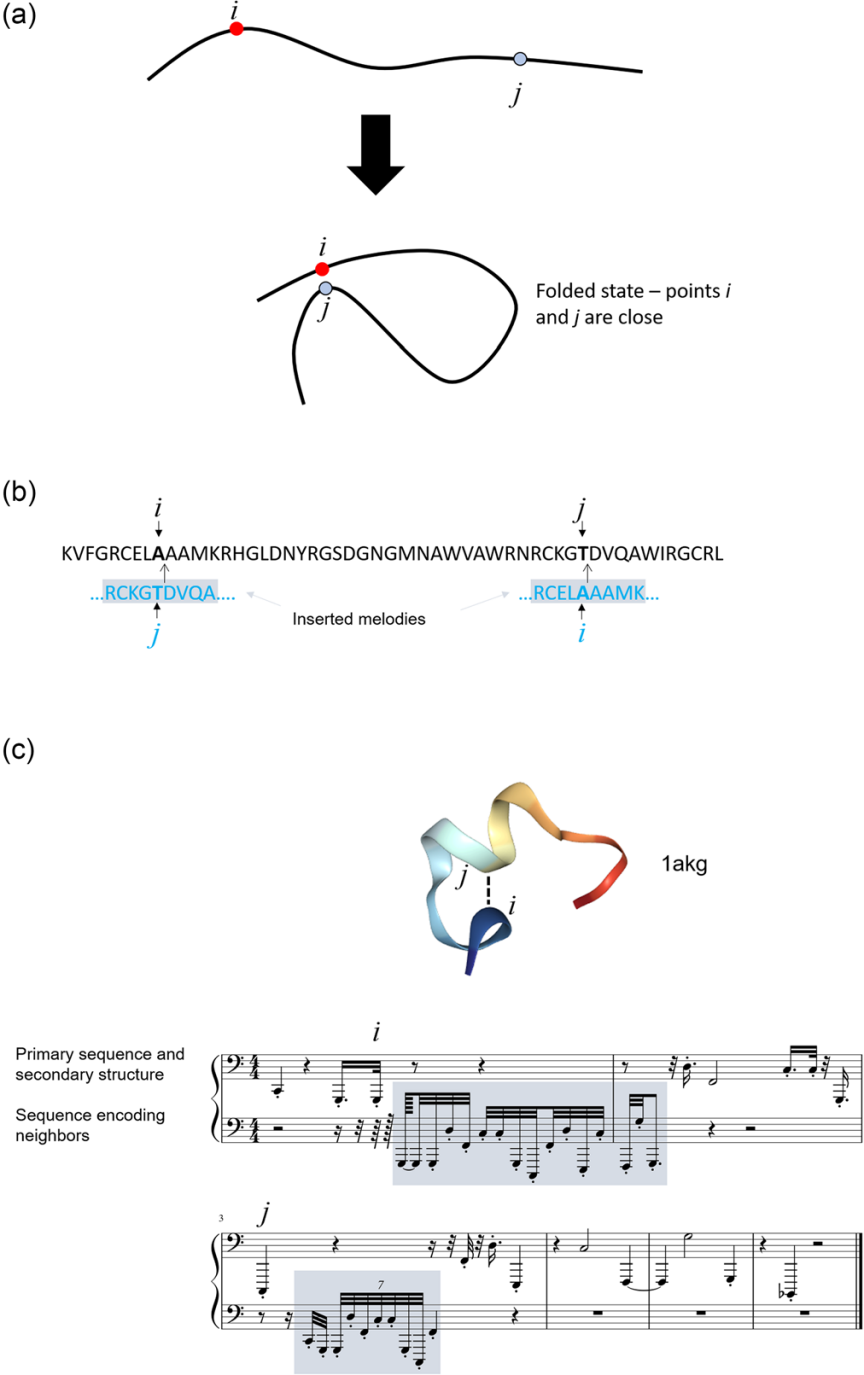
(a) Illustration of potential musical coding of protein folding, reflecting the incorporation of a higher order structure in music. The translation is achieved by reflecting the formation of close geometric interactions between different regions of the protein (points *i* and *j* in the example). (b) Illustration how the neighbors of points *i* and *j* (and vice versa) are coded, by marking a part of the sequence around *j* and inserting it near *i* in the musical score (and vice versa). This leads to overlapping melodies, and hence the playing of multiple notes at the same time (chords). By using an algorithm to find the pattern inserted near *i*, one can detect the location of its neighbor, making the method reversible. (c) Example of a musical score for protein with Protein Data Bank ID 1akg, reflecting a multi-track piece (upper—primary sequence, lower—inserted sequence patterns to code for folded geometry). 1akg_folded.mp3 is an audio file as an example (see supplementary material). Note, in this small protein, amino acid residue 3 is close to amino acid residue 9, and vice versa. Hence, there are two insertions in the music.

In future work, the sonification method could be further extended to address folded structures of proteins by including more spatial information, such as the relative distance of residuals, angles, or contact information into the audible signals. Figure 5(a) depicts an illustration of potential musical coding of protein folding, reflecting the incorporation of the higher order structure in music. The translation is achieved by reflecting the formation of close geometric interactions between different regions of the protein (points *i* and *j* in the example). Figure 5(b) shows how the neighbors of points *i* and *j* (and vice versa) are coded, by marking a part of the sequence around *j* and inserting it near *i* in the musical score (and vice versa). In musical notation, the inserted notes are played much faster and softer than the main sequence, with the note that reflects the amino acid of the neighbor played slightly louder. This coding in audible measures enables one to filter the relevant information from the notes played to ensure reversibility of the mapping. In fact, by using an algorithm to find the pattern inserted near *i* one can detect the location of its neighbor, making the method reversible. The reason why sequence patterns are inserted is to being able to detect—from matching the inserted sequence—which amino acid is the neighbor. Altogether, this approach leads to more complex melodies and depending on how quickly the inserted melodies are played, to chord progressions (similar to the strumming of a guitar). 1akg_folded.mp3 is an audio file as an example for how this new type of music sounds like (see the supplementary material), representing the score shown in Fig. 5(c).

Using this or similar approaches, one can directly translate between music notes and protein structure as long as the embedding information inside the protein-music mapping is self-consistent. Moreover, we do not have to change the structure of the deep neural network adopted in the present study because a different sonification method will only result in a different dataset that reflects the particular representation of music, which can be directly used to train the deep neural network. In preliminary testing, the representation of the folded structure of proteins as sketched in Fig. 5 has enabled us to use the deep neural network to generate musical patterns with identical structures as the training set, specifically creating musical scores that reflect the primary sequence with inserted melodies. 5xdj_seed_folded.mp3 is an audio file as an example for how a *de novo* protein generated from a 5xdj seed protein sounds like, whereas the inserted notes can clearly be identified by listening to the audio (see the supplementary material). Future work is needed to refine this approach, and it will be interesting to test whether the newly generated music is capable of correctly predicting the folding of the protein by analyzing the score.

The AI based approach to design new proteins opens the door to generative methods that can complement conventional protein sequence design methods. In future work, the method reported here can be further augmented by additional conditioning of the musical scores generated, and could also be combined with optimization algorithms such as genetic algorithms. New sequences predicted by the algorithm could be scored against performance measures and evolved further through the optimization method. This paper has focused on alpha-helical proteins. Future work could develop deep neural networks that include other protein motifs and perhaps additional and complementary classifications, so that more options for conditional generation can be developed.

## METHODS

### Translating protein sequences into musical scores

We map amino acid sequences into musical scores that reflect music composed in the “amino acid scale.” Using bioinformatics libraries Biopython and Biskit, we developed a python script that translates any sequence into a musical score. Sequences using the 1-letter amino acid code can be entered either manually or based on lists of one or more protein PDB identifiers. We also implemented a function by which proteins can be searched and grouped, using PyPDB. This allows one to quickly build complex musical scores for use as training sets for the neural networks. Musical scores are stored as MIDI files that are used for the training.

To reflect a higher-order chemical structure in the musical representation, we incorporate information about the secondary structure associated with each amino acid in the translation step in affecting the duration and volume of notes. We use DSSP to compute the secondary structure from the protein geometry file and sequence.^29,30^ Table II lists the complete set of parameters determined by this approach. We use longer note durations for disordered secondary structures, very short note durations for helices, and short notes for beta-sheets. We also modulate the volume by rendering beta-sheets the loudest, and others more softly. For instance, ALA residues in a BS will be played loud and slower than ALA residues in an AH, which will be played in a fast, repetitive manner. Similarly, ALA residues in random coils or unstructured regions are played slowly and softly. These modulations of the tone by volume and timing lead to a certain rhythmic character that overall reflects the 3D folded geometry of the protein. For the training of the neural networks, capturing these features is essential, as it reflects the hierarchical nature of the protein fold from primary, secondary, to tertiary, and higher-order structures.

**TABLE II.** Incorporation of the secondary protein structure in the translation into musical score, affecting note timing and note volume. Each of the 20 amino acids is mapped into a distinct musical note, from C-2 to A1, on a C major scale (the white keys on a piano). Additional musical features are introduced by mapping higher-order structural information, as summarized in the table. Different amino acid chains within one protein are characterized by a B-2 note and a longer break, and different proteins are characterized by a A#-2 note and even longer break. By classifying three major secondary structure classes, we can capture their representation in musical space, and also translate the feature into the AI. The variations in pitch, volume, and note timing introduce musical characteristics that represent the hierarchical protein structure in that space.

Secondary structure	Note assignment	Note duration (normalized by a 1/8th note; i.e. there are 8 notes per bar)	Note volume (normalized by reference MIDI note volume = 100)
Beta-sheet (all types)	N/A (notes assigned according to mapping of 20 amino acids into notes, see Fig. 6)	1.0 (i.e., 1/8th notes)	1.0
Helices (alpha helix and others)	N/A (notes assigned according to mapping of 20 amino acids into notes)	0.5 (i.e., 1/16th notes)	0.5
Random coil and unstructured	N/A (notes assigned according to mapping of 20 amino acids into notes)	2.0 (i.e., 1/4 notes)	.25
Separation between different amino acid chains	B-2 marked as ! in predicted amino acid sequences	4.0 (long break to indicate new amino acid chain)	1.0
Separation between different proteins	A#-2 marked as # in predicted amino acid sequences	8.0 (long break to indicate new protein)	1.0

### Deep neural network and training

**FIG. 6.**
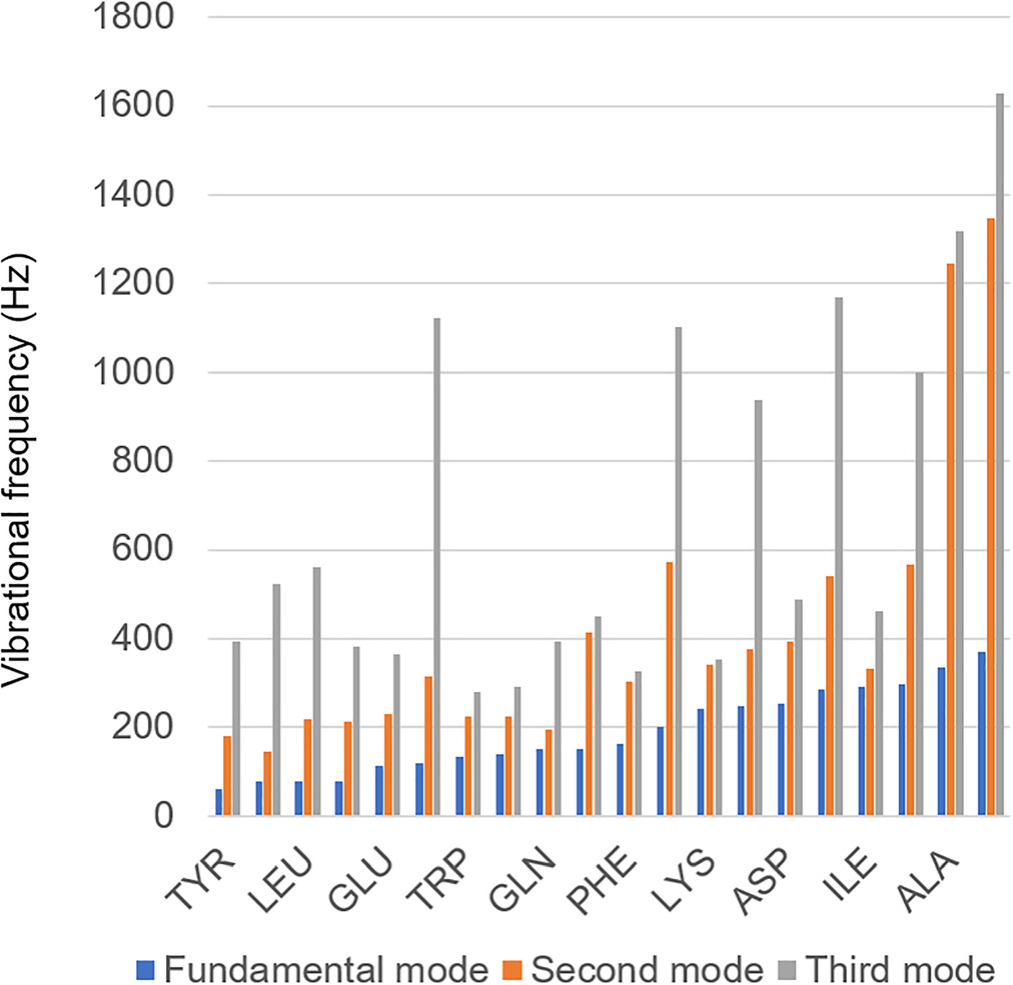
First here vibrational frequencies associated with each of the 20 amino acids (fundamental frequency and second and third higher modes). Original data for the plot shown taken from Ref. 31, full analysis of audio spectrum in Ref. 20, whereas the vibrations derived from density functional theory (DFT) are transposed to the audible frequency spectrum using the music theoretical concept of transpositional equivalence.^47^ For analysis of the data in conventional music software, each of the 20 amino acids is mapped into a distinct musical note, from C-2 to A1, on a C major scale (the white keys on a piano). This representation is used in the sample musical score of one of the proteins in the training set shown in Fig. 7. It is noted, however, that the sound of each protein is not within any conventional musical scale. Rather, the soundings of the amino acids create a unique associated “amino acid scale.”

**FIG. 7.**
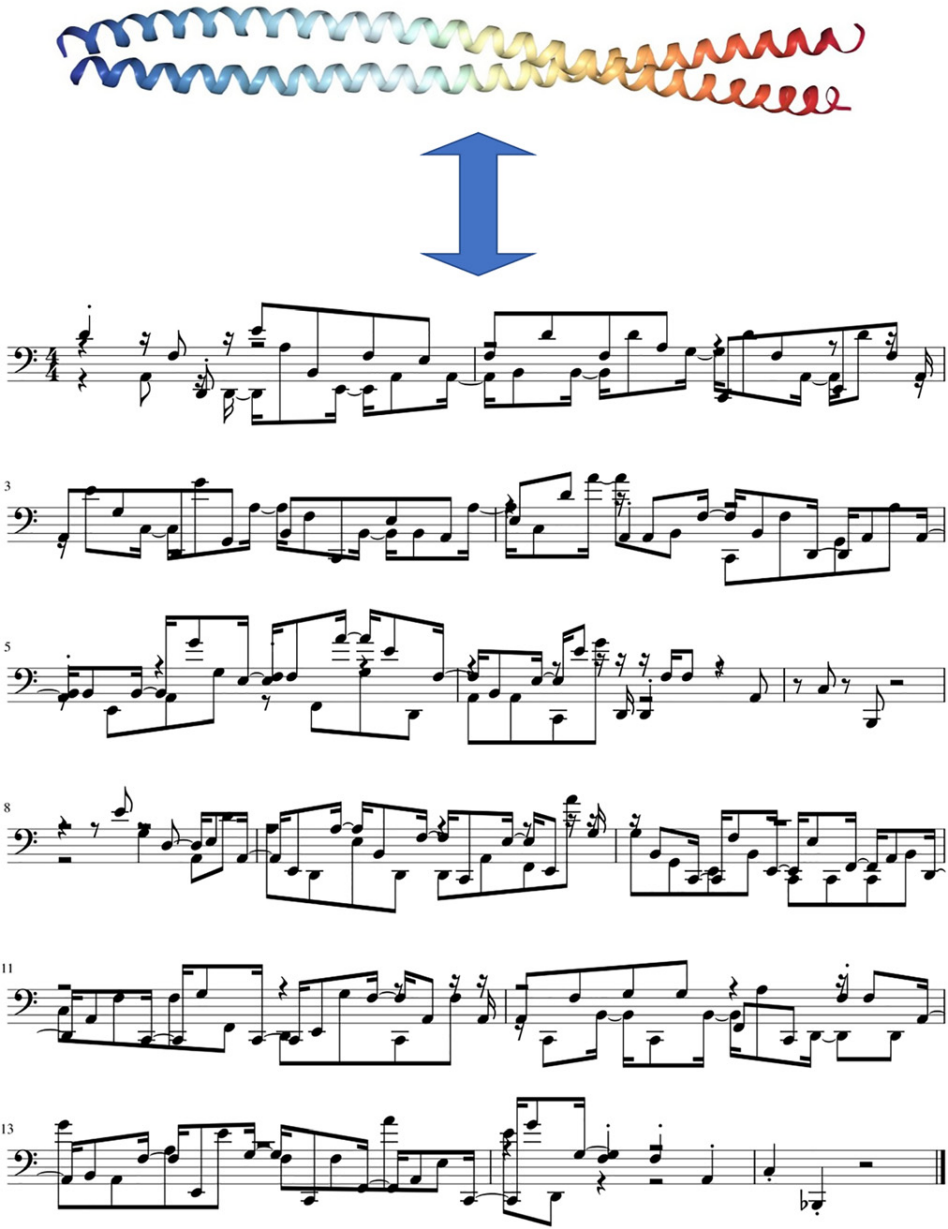
Musical score generated for the protein 3tnu (intermediate filament protein), part of the training set. The musical score is 14 bars long. Note that the notes indicated do not reflect a conventional musical scale, but that each note in the space of 20 admissible tones in the native amino acid scale is assigned to one of the 20 amino acids. The score is shown here only for visualization of the concept, and to illustrate the timing, rhythm, and progression of notes as learned from the amino acid sequence. A large number of musical scores are used as the training set to train the neural network.

The deep neural network model is formulated based on the concept of using a translation of protein structures into musical space, reflecting the 20 amino acids and secondary structures as distinct and reversible audible expressions. A summary of the mappings from the protein structure to musical scores is summarized in Table II. The basis to mapping each of the 20 amino acids onto a unique musical tone is the unique vibrational spectrum of the molecules as explained in Ref. 26, each of the amino acids has a unique vibrational spectrum, which allows us to distinguish each of them by its unique sound (or timbre). Figure 6 summarizes the lowest vibrational frequencies (first harmonic and two higher^31^) of each of the 20 amino acids in ascending order, showcasing the unique vibrational characteristic. For analysis of the data in conventional music software, each of the 20 amino acids is mapped into a distinct musical note, from C-2 to A1, on a C major scale (the white keys on a piano). This representation is used to create musical scores. An example of one of the proteins in the training set is shown in Fig. 7, showing the musical score of the protein with PDB ID 3tnu (an intermediate filament protein).

We translate a set of alpha-helix rich proteins into musical scores and use this representation to train a deep neural network, using the Magenta framework.^32^ The recurrent neural network (RNN) we employ in this work is adopted from language modeling, implemented in the Performance RNN model^33,34^ using TensorFlow.^35^ This RNN layers Long Short-Term Memory Units (LSTM) for time sequence features, alongside a dynamical conditioning.^36^ The attention dynamical conditioning model is able to monitor the note velocity changes of the note sequences, which is important to capture a higher-order structure of proteins. We use a batch size of 64, and three layers with sizes 512, 512, and 512. We use the “performance_with_dynamics” model to model note pitches, note timing, and note velocity changes. Training is done until convergence is achieved, typically around 100 000 steps. The training and generations are done on a Dell Precision Tower 7810 workstation (Xeon CPU E5–2660 v4 2.0 GHz, 32 G memory with a GeForce RTX 2080 Ti GPU).

#### *Training set: Alpha-helix rich proteins collected from the Protein Data Bank (PDB;* https://www.rcsb.org/ *)*

We use a training set consisting of alpha-helix rich proteins (PDB IDS: 6A9P, 6F62, 6F63, 6F64, 6GAJ, 6GAK, 5VR2, 5TO5, 5TO7, 5XDJ, 5LBJ, 2NDK, 5WST, 5IIV, 5D3A, 5HHE, 2MG1, 2LBG, 2L5R,3V4Q, 2D3E, 2HN8, 2FXO, 3TNU, 4YV3, 1GK6, 3SSU, 3SWK, 2XV5, 3UF1, 3PDY, 1X8Y, 3TNU, 4ZRY, 6E9R, 6E9T, 6E9X, 2MG1; a total of around 20 000 amino acid residues).

The protein sequences are translated to musical scores using the method described in Table II, recorded in MIDI format, and then used for the neural network training.

### Generation of new musical scores

Once the deep neural network described in the previous section is trained properly, we use it to generate new protein sequences. The neural networks will produce musical scores in the MIDI format, which are mapped back into protein sequences using a Python script. Different musical scores are used as a primer to seed the generation process. We use either musical scores reflecting amino acid sequences of entire existing protein structures or use short note sequences corresponding to brief patterns of amino acid sequences. Varying the seed enables us to generate different musical scores.

On the other hand, we use temperature, a hyperparameter of the deep neural network, during the generation process to tune the generated musical scores. This parameter affects the randomness of predictions. The use of temperature is a common way to achieve control of the generated output. The baseline of temperature is set to 1.0. In this case, the probability distribution we used to generate the AA sequence is initiated according to the training directly. One can control the randomness by manipulating temperature. We can reduce the randomness of probability distribution by decreasing the temperature value (T<1); or introduce more randomness by introducing a higher value of temperature, say T>1.

We find that using temperature values in the range from 1.0 to 2.0 results in good predictions, whereas a value closer to 1.0 yields amino acid sequences closer or identical to patterns found in the training set and a value well in excess of 1.0 yields sequences that are distinct from any amino acid sequences of the training set. We also discover that the predicted musical scores contain the same musical notes as the training scores, and that similar musical patterns of volume and timing variations are generated. The higher the temperature and the smaller the length of the seed note sequence, the greater the variations from the training set. For higher temperature choices (in excess of 2.0), some generated notes fail to fall on the set of 22 notes used in the training set, reflecting the greater variations and error introduced (this serves as a guideline to maintain the temperature values in the range from 1.0 to 2.0). Further studies could explore more variations of the interplay of temperature and seed musical score on the predicted proteins.

### Mapping musical scores back into protein sequences

To map musical scores back into amino acid sequences, we developed a script that reads a Musical Instrument Digital Interface (MIDI) file and maps the notes associated with the 20 amino acids back onto amino acids, generating sequence outputs in the 1-letter codes. In the translation of the musical scores back into amino acid sequences, we solely capture the *sequence* of amino acids. This serves as a means to test the predictive power of the neural networks as to whether or not they are capable of predicting proteins with the desired secondary structures. In principle, secondary structure information could be extracted from the musical scores as well (in this paper, we use it as a validation step to confirm that the musical structure agrees with the folded protein structure, as explained in the Results section below).

### Amino acid sequence analysis

Sequence similarities are analyzed using BLAST.^37^ We use the blastp (protein-protein BLAST) algorithm for the examples discussed in this paper. We assess various scoring functions in the analysis, specifically query cover, percent identical, and the overall Max score.

### Protein structure prediction

The sequence data generated by the neural network are used for further analysis to examine similarities with known proteins and then used to build 3D models using protein folding methods. We use a homology method, ORION,^38^ to predict an estimated structure of the designed protein sequences. A 3D structure is obtained using MODELLER,^39^ reflecting the images shown in Table III (right column). We use the PDB95 database in ORION, representing a collection of 54 540 protein templates, gloloc alignment mode, and consider up to 100 proposed structures. The top structure with the highest score is used for further analysis and shown in the table.

**TABLE III.** Summary of AI designed *de novo* proteins using the neural network model developed. The name of the identifiers of the proteins corresponds to the PDB ID as listed in https://www.rcsb.org.

Seed and temperature, notes on predicted sequence	Image of folded protein generated by AI
5xdj, temperature = 1.2, 3000 steps	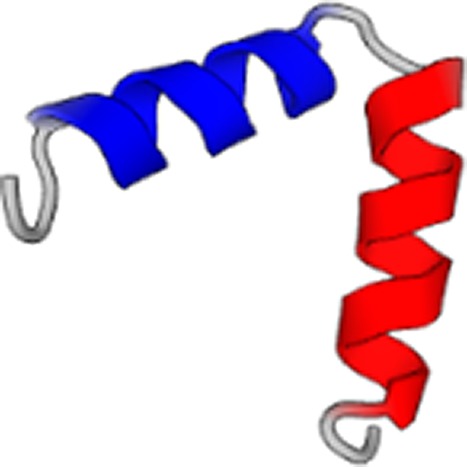
5xdj, temperature = 1.4, 3000 steps	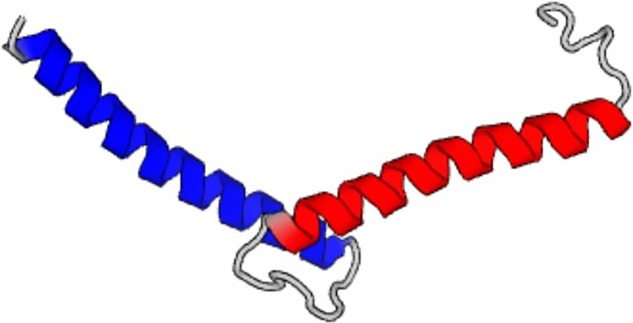
5xdj, temperature = 1.8, 3000 steps	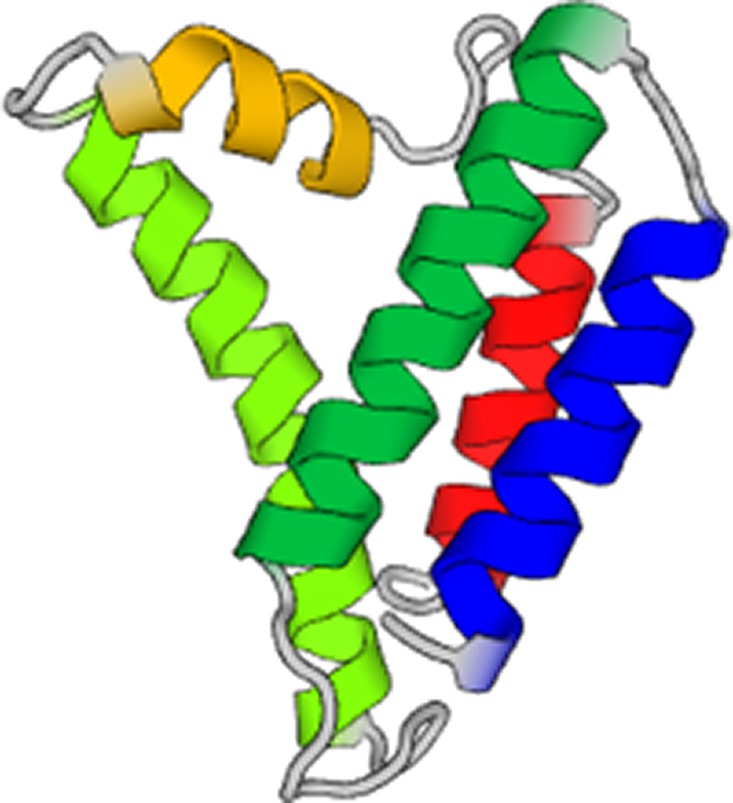
Resulting sequence shows some similarities with PDB ID 9e9x	
5xdj, temperature = 1.8, first amino acid fragment that is *de novo* and not part of the training set nor any databases	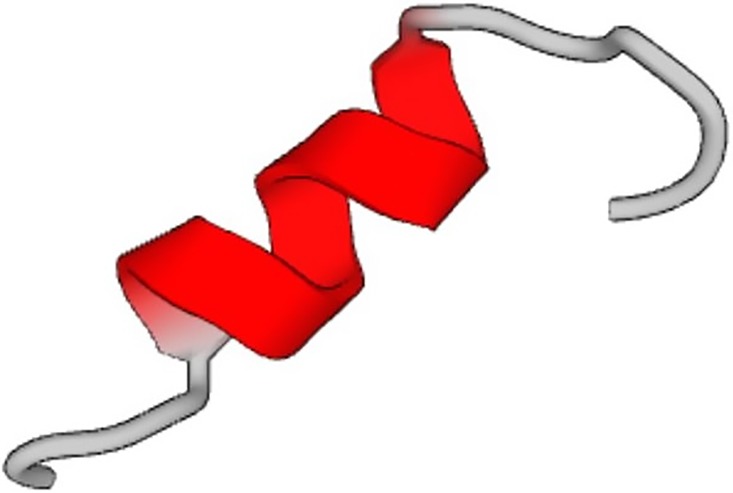
SEVELQRALEIARESGT	
5xdj, temperature = 2.0, 10 000 steps	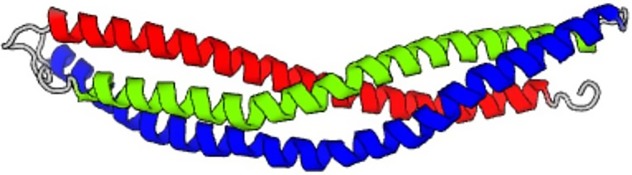
First sequence marked in red in the text	
4xdj, temperature = 2.0, 10 000 steps	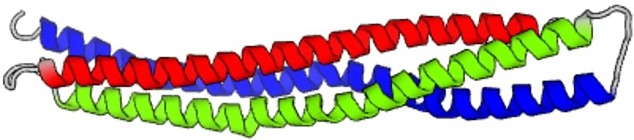
Second sequence marked in green in the text	
2ndk, temperature = 1.2, 3000 steps	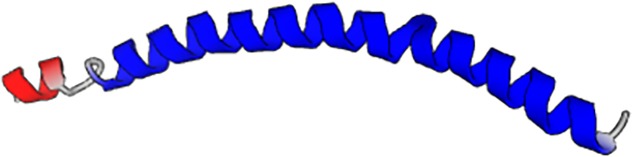
2ndk, temperature = 1.2, 3000 steps,	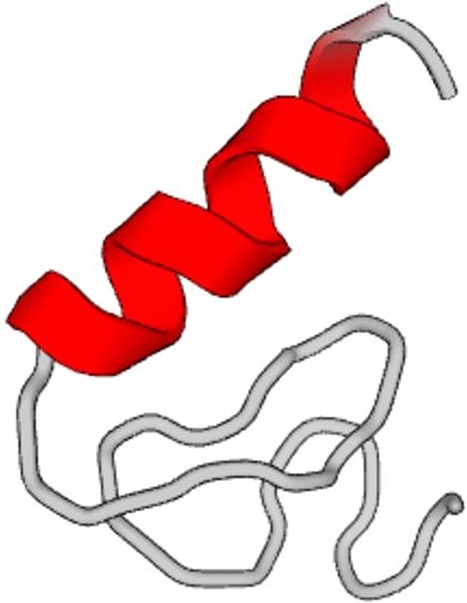
Predicted structure of the first sequence pattern that is *de novo* and not part of the training set nor any databases	
EHEAERRDKNKLTAETEKGIMAYMAFLKEAERRSDEGQTNTVTL	

In addition to using the aforementioned method, we also used I-TASSER^40,41^ to compare against the ORION predictions. We carried out this computation for select sequences (specifically, the predictions for the 5xdj seed, temperature = 2.0, 10 000 steps) and confirmed the emergence of alpha-helical proteins. The I-TASSER predictions tend to include elongated alpha-helical structures rather than the folded coiled-coil like geometries seen in ORION. However, it is seen that the alpha-helical domains have very short “breaks” that may effectively act as hinges, ultimately leading to the self-folding of the structure as suggested in Ref. 42. The I-TASSER results also include information about the possible protein function, including ligand binding sites and ligand names, as well as active sites.

### Molecular dynamics modeling

We use NAMD (implemented in CUDA for execution on a GPU; version: NAMD_2.13_Linux-x86_64-multicore-CUDA)^43^ with the CHARMM force field^44^ to minimize and equilibrate protein structures at 310 K, using a Langevin thermostat. Visual Molecular Dynamics (VMD)^45^ is used for pre- and post-processing and image and movie generation. An explicit water solvent is modeled using TIP3P water. The simulations are carried out at neutral pH and 0.15 mol/l NaCl concentration. The molecular simulations are done on a Dell Precision Tower 7810 workstation (Xeon CPU E5–2660 v4 2.0 GHz, 32 G memory with a GeForce RTX 2080 Ti GPU.

### Normal mode analysis

We conduct a normal mode analysis using an anisotropic network model^46^ of the protein PDB generated based on AI, using the equilibrated protein structure as input.

## AUTHOR'S CONTRIBUTIONS

M.J.B. designed this research, in collaboration with C.-H.Y. The paper was written by M.J.B. with input from C.-H.Y. All authors participated in all aspects of the research.

## SUPPLEMENTARY MATERIALS

See the supplementary material for equilibration of the *de novo* protein predicted using the recurrent neural network, in explicit water (file accessible in Data Sheet 1.ZIP) (Movie M1 PDB1-equilibration.mpg); PDB File of the equilibrated protein structure (file accessible as Video 1.MPG) (PDB1.pdb); audio file of the sonification of the *de novo* protein designed here, as shown in Fig. 3 (PDB1_sonified.mp3); audio file reflecting the sonification of a folded protein with Protein Base identifier 1akg, using overlapping melodies to code for the overall 3D structure (as sketched in Fig. 5); in this example, the inserted notes reflecting the amino acid sequence of the geometric neighbor are added as inserted melodies, embedded within the primary sequence (1akg_folded.mp3); and audio file as an example for how a *de novo* protein generated from a 5xdj seed protein; the inserted notes, played in rapid succession, can clearly be identified, showing that a deep neural network trained based on a large set of musical scores with folding information generates the appropriate types of scores (5xdj_seed_folded.mp3).
